# Controlling All‐Optical Helicity‐Dependent Switching in Engineered Rare‐Earth Free Synthetic Ferrimagnets

**DOI:** 10.1002/advs.201901876

**Published:** 2019-10-14

**Authors:** Jung‐Wei Liao, Pierre Vallobra, Liam O'Brien, Unai Atxitia, Victor Raposo, Dorothée Petit, Tarun Vemulkar, Gregory Malinowski, Michel Hehn, Eduardo Martínez, Stéphane Mangin, Russell P. Cowburn

**Affiliations:** ^1^ Cavendish Laboratory University of Cambridge J J Thomson Avenue Cambridge CB3 0HE UK; ^2^ Institute Jean Lamour UMR CNRS 7198 Universite de Lorraine 2 allée André Guinier‐BP 50840 54011 Nancy France; ^3^ Department of Physics University of Liverpool Liverpool L69 7ZE UK; ^4^ Department of Physics Freie Universität Berlin Arnimalle 14 14195 Berlin Germany; ^5^ Department of Applied Physics of the Faculty of Science University of Salamanca 37008 Salamanca Spain

**Keywords:** all‐optical switching, ferromagnets, synthetic ferrimagnets

## Abstract

All‐optical helicity‐dependent switching in ferromagnetic layers has revealed an unprecedented route to manipulate magnetic configurations by circularly polarized femtosecond laser pulses. In this work, rare‐earth free synthetic ferrimagnetic heterostructures made from two antiferromagnetically exchange coupled ferromagnetic layers are studied. Experimental results, supported by numerical simulations, show that the designed structures enable all‐optical switching which is controlled, not only by light helicity, but also by the relative Curie temperature of each ferromagnetic layer. Indeed, through the antiferromagnetic exchange coupling, the layer with the larger Curie temperature determines the final orientation of the other layer and so the synthetic ferrimagnet. For similar Curie temperatures, helicity‐independent back switching is observed and the final magnetic configuration is solely determined by the initial magnetic state. This demonstration of electrically‐detected, optical control of engineered rare‐earth free heterostructures opens a novel route toward practical opto‐spintronics.

## Graphical abstract

All‐optical helicity‐dependent switching in synthetic ferrimagnetic samples made of two exchanged coupled ferromagnetic layers is shown to depend on the respective Curie temperature of each layer. By varying the CoFeB layer thickness, its Curie temperature can be tuned. Consequently, the switching is set not only by the light helicity but also by the CoFeB thickness.

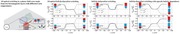

## 1 Introduction

Controlling magnetic state without the application of a magnetic field draws substantial research interest, particularly as a means to avoid localized magnetic field generation within magnetic logic and data‐storage devices. One method for magnetic reversal that holds great promise is the use of circularly polarized optical laser pulses:^1^ Spurred by the first observation of all‐optical switching (AOS) in rare earth (RE)‐transition metal (TM) alloys, such as GdFeCo,^2^ all‐optical helicity‐dependent switching (AO‐HDS) represents a fast and facile method to manipulate magnetization. AO‐HDS has been observed in a high number of materials, and the term AOS now encompasses several possible microscopic mechanisms for controlling magnetization with polarized light pulses. These range from the helicity‐independent switching (AO‐HIS) seen in several Gd‐based samples, for example, GdCo alloys—arising from picosecond‐duration angular momentum transfer between the two antiferromagnetically (AF) oriented sublattices in RE‐TM alloys^3^—through to AO‐HDS in RE‐free ferromagnets (FMs),^4^ such as Pt/Co/Pt multilayers,^5^ where switching occurs even in the absence of AF coupling. In this case, both laser‐induced magnetic domain nucleation and domain‐wall (DW) motion^4^, ^5^, ^6^, ^7^ are shown to play an important role.

Despite the numerous systems where AO‐HDS has been observed, in all cases any helicity dependence is intrinsic to the material composition under investigation and cannot be tuned, limiting both our ability to understand the mechanisms at play during reversal, and their ultimate use in technological applications. Synthetic ferrimagnets (SFi), where a composite ferrimagnet is fabricated from two (or more) antiferromagnetically (AF) exchange coupled thin film FM layers, are a potential solution to this issue.^8^, ^9^ In SFi's, the interfacial AF coupling and the thermal response of the magnetization (i.e., nucleation of domains) within each FM layer can be tailored independently,^10^ and so, in principle, the AO‐HDS mechanism. Within the wider class of composite SFi materials, RE‐free TM/FM multilayers^8^ are particularly appealing due to the low cost and earth abundance of their constituent materials. The role of magnetic anisotropy^11^ and a magnetic compensation temperature^8^ are closely scrutinized to understand AO‐HDS in this material class, while the underlying mechanism driving AO‐HDS in RE‐free SFi's is still debated. To this end the full control of a given composite material, for example, modulating the HD, is yet to be achieved.

**Figure 1:**
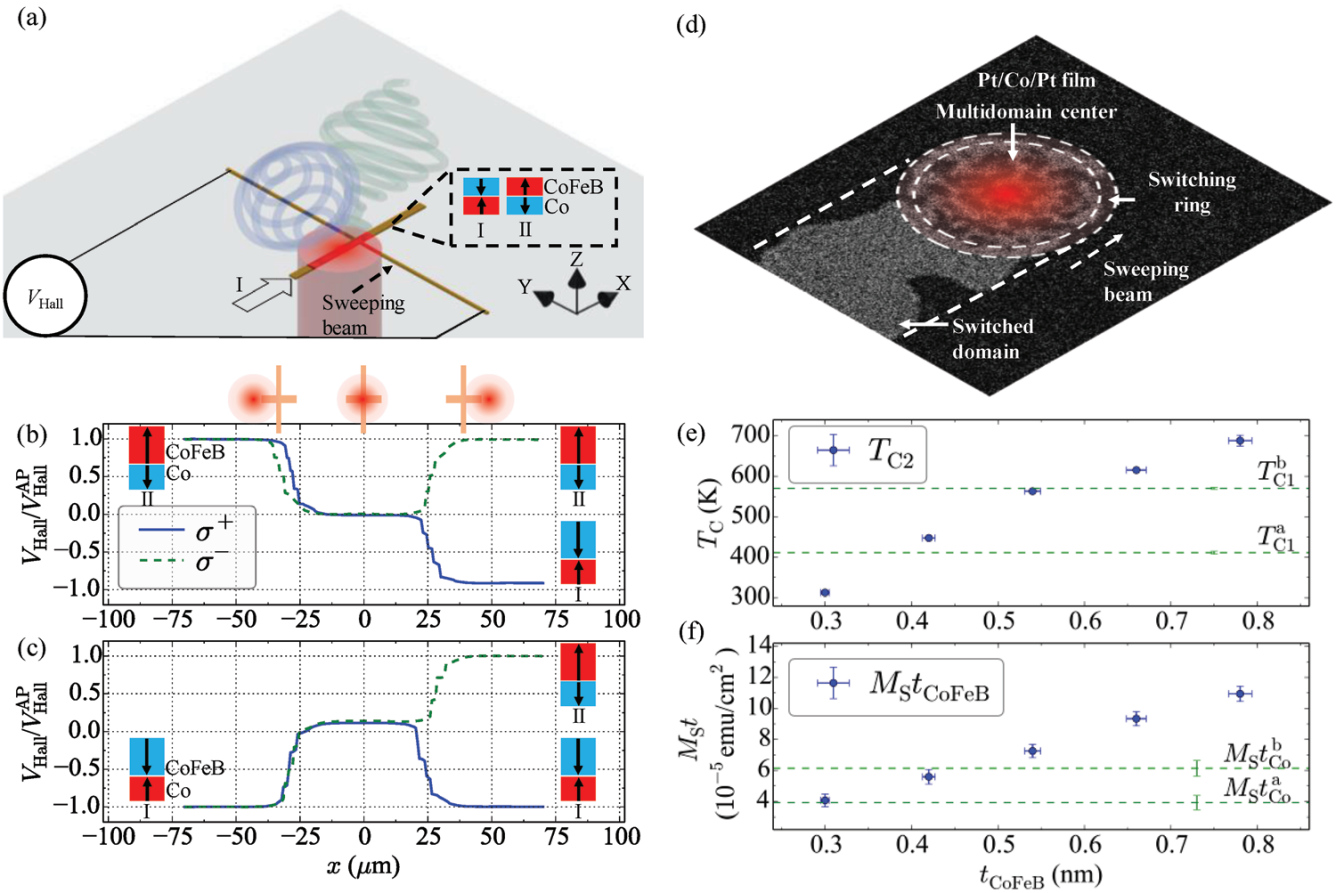
a) Schematics of the laser sweeping measurement, with initial State I and II shown. b,c) Measured normalized Hall voltage, *V*_Hall_/*V*_Hall_^AP^, versus beam position, *x* (relative to cross center) for an SFi Hall bar with *t*_Co_ = 0.73 nm and *t*_CoFeB_ = 0.54 nm. Initial magnetic state is (b) State II and (c) State I. The measurement is repeated with two different circular polarizations, σ^+^ (solid line) and σ^−^ (dashed line). d) Magneto‐optical Kerr image of a single Pt/Co/Pt film during the laser beam sweep. e,f) Variation of Curie temperature, *T*_C_, and the magnetization per unit area, *M*_S_ *t*, as a function of the FM2 layer thickness. The horizontal lines indicate the properties of the Co layer. *T*_C1_^a^ corresponds to *t*_Co_^a^ = 0.49 nm, and *T*_C1_^b^ to *t*_Co_^b^ = 0.78 nm.

In this paper, we demonstrate direct control of AO‐HDS in RE‐free SFi's through manipulation of the Curie temperature, *T*_C_. AO‐HDS due to irradiation by circularly polarized femtosecond laser pulses is investigated using anomalous Hall effect (AHE) measurements of SFi microbars (**Figure** **1**a).^12^ Through experiment and temperature‐dependent micromagnetic simulation we determine that the reversal of the SFi is set by the distinct thermal response of the two coupled magnetic layers. A model prevails where the laser fluence heats the FM layer with the lower *T*_C_ above its *T*_C_, while the helicity sets the magnetization direction of the other FM layer, through a mechanism such as the inverse Faraday effect (IFE) acting as a magneto‐optical field on the FM layer,^13^ breaking the symmetry and so causing AO‐HDS. Ultimately, by altering the thermal response of each layer, through modification of the individual layer *T*_C_, we demonstrate a simple means to achieve all‐optical control of magnetic heterostructures composed of commonly used materials, which may provide a promising route to practical opto‐spintronics.^14^

## 2 Results and Discussion

We perform measurements on micrometer‐scale SFi Hall bars consisting of two perpendicularly magnetized, AF‐coupled FM layers, termed FM1 (Co/Pt) and FM2 (CoFeB/Pt/CoFeB) and with variable thickness *t*_Co_ and *t*_CoFeB_. Varying thickness allows us to control the *T*_C_ of each layer^15^ and therefore thermal response. Vibrating sample magnetometry (VSM) confirms two possible AF remnant states (see Figure 1a; State I (II), FM1 points in +*z* (−*z*)), with magnetization, *M*, of each FM uniformly aligned out‐of‐plane and antiparallel to one another (Section S1, Supporting Information). In the SFi Hall bars, AHE measurements, *V*_Hall_ (Section S1, Supporting Information) provide an electrical measurement sensitive to *M*_z_ of each layer, and confirm remnant states I and II are maintained in the patterned films.

Figure 1a illustrates the sweeping laser beam method used to investigate the response of the SFi to fs light pulses (discussed in the Experimental Section). The SFi is initialized in a known state (I or II) before pulsed, circularly polarized laser light (either σ^+^ and σ^−^), focused to an ≈46 µm spot, is swept along the *x*‐axis arm. *V*_Hall_ is monitored as the laser passes over the Hall cross. Figure 1b (initial state I) and (c) (initial state II) illustrate the resulting *V*_Hall_ versus relative beam position, *x*, for each incident polarization, and normalized to the remnant value, *V*_Hall_^AP^_,_ for *t*_Co_ = 0.73 nm and *t*_CoFeB_ = 0.54 nm. Here, one can clearly see that *V*_Hall_ decreases toward zero as the laser beam becomes centered on the cross. This is attributed to an initial helicity‐independent demagnetization, as observed in some SFi's and single FM layers,^12^ resulting from the central portion of the laser heating FM1 and FM2 above *T*_C_, causing loss of long‐range order. With no locally favored state (I or II), upon cooling the irradiated film reorders multidomain. This demagnetized state is stable, persisting after removal of the laser beam, and so is presumed to occur only when *T* > *T*_C_ for both individual layers, *T*_C1_ and *T*_C2_. As the laser spot passes the cross a helicity‐dependent remagnetization occurs, that is, net magnetization is restored, with the σ^+^ beam switching to final State I, while σ^−^ polarized beam results in State II. It is apparent from this behavior that AO‐HDS occurs in these SFi's not within the beam center (which remains demagnetized), but at the trailing edge of the beam profile. Changing the initial state from State II to State I, in Figure 1c we observe the same dependence of the final state on laser helicity, demonstrating both AO‐HDS in RE‐free SFi's and an independence of the mechanism on initial state.

On first inspection, the composite SFi appears to behave as a single FM layer (such as Pt/Co/Pt):^12^ Provided the beam fluence is sufficiently large, *T* > *T_C_* and thermal demagnetization occurs, with multiple domains nucleated. A ring around the central region exists where *T* < *T*_C_ (so the film remains magnetized), but exceeds the threshold for AO‐HDS.^7^ On sweeping the beam, the helicity favors one magnetization direction over the other, remagnetizing the SFi (Figure 1d). While this argument qualitatively explains data presented thus far, and follows the prevailing wisdom for single FM reversal, the situation must be more complex for SFi's where *T*_C1_ ≠ *T*_C2_, as is the case here. For these materials, while the central beam may still demagnetize the bar, a region exists where, for example, *T*_C1_ < *T* < *T*_C2_, that is, FM2 remains magnetized while FM1 has lost long‐range order. Should this also be sufficiently high that AO‐HDS can occur, within this ring the laser fluence and so any HD effects (such as magnetic circular dichroism (MCD), or the inverse Faraday effect (IFE)) now act not on the composite SFi as a whole, but on an individual FM layer (in this case FM2). Consequently, AO‐HDS is intimately linked with the precise ordering of the SFi on cooling, and tuning each *T*_C_ offers the opportunity to tailor any helicity dependence.

To test this hypothesis, we independently vary *T*_C_ in each layer. To minimize the effect of changing other parameters, we maintain the same materials for FM1 and FM2 and instead tune *T*_C_ by varying FM thickness. Figure 1e,f shows the resulting variation in (e) *T*_C2_ as a function of *t*_CoFeB_, and (f) *M*_s_ *t*_CoFeB_, the layer moment per unit area. Reducing *t*_CoFeB_ reduces *T*_C2_ from bulk, due to a transition toward 2D magnetic behavior,^15^ while also linearly decreasing *M*_s_ *t*_CoFeB_ (i.e., with minimal changes to *M*_S_). For comparison, in Figure 1e,f *T*_C1_ and *M*_s_ *t*_Co_ are shown for two different tested thicknesses of FM1, *t*_Co_ = 0.49 nm and *t*_Co_ = 0.73 nm. Examining the two plots, one can easily see for a given *t*_Co_ the critical FM2 thicknesses where *T*_C2_ exceeds *T*_C1_, which in the case of *t*_Co_ = 0.73 nm occurs above *t*_CoFeB_ ≈ 0.6 nm.

**Figure 2:**
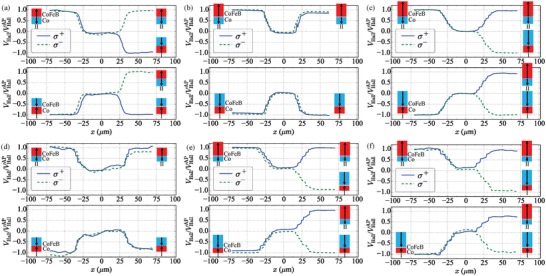
a–c) Variation of the normalized Hall voltage, *V*_Hall_/*V*_Hall_^AP^(*x*), for SFi with *t*_Co_ = 0.73 nm and varying *t*_CoFeB_. (a) *t*_CoFeB_ *=* 0.42 nm, (b) 0.66 nm, and (c) 0.78 nm. Initial State I (bottom panel) or State II (top). The measurements are repeated using σ^+^ (solid line) and σ^−^ (dashed) polarization. d–f) *V*_Hall_/*V*_Hall_^AP^(*x*), for SFi with *t*_Co_ = 0.49 nm and varying *t*_CoFeB_. (d) *t*_CoFeB_ *=* 0.42 nm, (e) 0.66 nm, and (f) 0.78 nm.

Investigating AO‐HDS in this range, shown in **Figure** **2**a–c for *t*_Co_ = 0.73 nm, a pattern emerges: At low *t*_CoFeB_ (e.g., *t*_CoFeB_ < 0.66 nm, Figure 2a and Section S2, Supporting Information), that is, while *T*_C1_ > *T*_C2_, we observe a similar AO‐HDS dependence as Figure 1b,c, with σ^+^ light remagnetizing the SFi toward State I. Moving to the point where *T*_C1_ ≈ *T*_C2_ (Figure 2b, *t*_CoFeB_ = 0.66 nm) a complete loss of AO‐HDS is surprisingly found, with helicity‐independent reversion to the initialized state. Finally, further increasing *t*_CoFeB_ until *T*_C1_ < *T*_C2_, for example, for *t*_CoFeB_ = 0.77 nm (Figure 2c) restores AO‐HDS, however, now the helicity dependence is inverted and the σ^+^ beam leads to final State II. Through tuning the *T*_C_ within each layer, we demonstrate direct control of AO‐HDS.

We may compare these results against samples with reduced *t*_Co_ = 0.49 nm. Here, *T*_C1_ ≈ *T*_C2_ occurs at lower FM2 thickness (*t*_CoFeB_ ≈ 0.4 nm) which, in theory, should be reflected in any crossover of HD. Figure 2d–f shows the resulting *V*_Hall_/*V*_Hall_^AP^(*x*) for *t*_CoFeB_ = 0.42, 0.66, and 0.78 nm, respectively. Again, when *T*_C1_ = *T*_C2_ (Figure 2d, *t*_CoFeB_ ≈ 0.42 nm) helicity‐independent reversion occurs, while for *T*_C2_ > *T*_C1_ identical AO‐HDS is found for all samples, with σ^+^ leading to final State II (Figure 2e,f and Section S2, Supporting Information).

These results cannot be understood by current models of AO‐HDS in single FMs, where switching is intrinsically set by the light helicity and is *T*_C_ independent.^5^ Here, we propose a scenario to account for the observed *T*_C_ dependence: When irradiating an SFi, for example, with *T*_C1_ > *T*_C2_, due to the continuous *T* distribution across the beam, a narrow ring exists where *T*_C1_ > *T* > *T*_C2_ and FM1 remains ordered, but demagnetized, while FM2 loses any long‐range order. AO‐HDS occurs in FM1 throughout the ring, without the presence of any interlayer exchange coupling (since *M*_2_ = 0), and the layer begins to remagnetize. As the laser pulse is removed and the sample cools, the reordering of FM2 and restoration of AF coupling forces FM2 antiparallel to FM1: The SFi ordering is dictated by FM1. Should *T*_C2_ > *T*_C1_, the situation becomes reversed and the helicity dependence of FM2 dictates ordering of the SFi. When *T*_C1_ ≈ *T*_C2_ the ring width collapses, no domain orientation direction favored (the helicity favors FM1 and FM2 moments to be parallel, while the AF coupling favors those moments to be antiparallel), and no, or at least weak, AO‐HDS occurs. Simple checks of the reversal symmetry of the SFi stack with *T*_C1_ > *T*_C2_, as compared with single Co FM layers (*t*_Co_ = 0.73 nm, see Section S3, Supporting Information), confirm a consistent helicity dependence between the two. Furthermore, measurements replacing the circular polarization with linearly polarized light and an externally applied field (*H*_Z_ ≈ 3 Oe) shows an equivalent symmetry to AO‐HDS (see Section S4, Supporting Information). We interpret the interaction of the circularly polarized light with *M* to be equivalent to an effective field in this case, potentially as a result of the IFE.^13^

**Figure 3:**
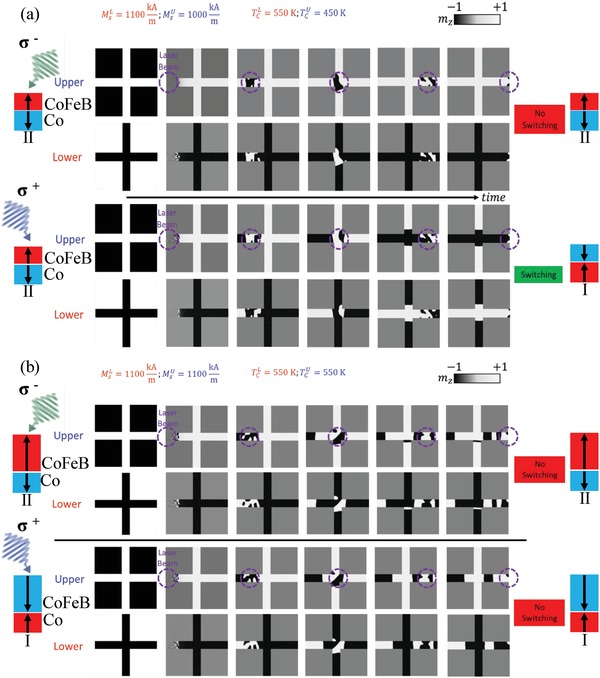
Modeled transient snapshots of magnetic configurations in the Hall crosses. The crosses are composed of two ferromagnetic layers with a) *M*_S_^Co^ < *M*_S_^CoFeB^ and *T*_C_^Co^ < *T*_C_^CoFeB^ and b) *M*_S_^Co^ = *M*_S_^CoFeB^ and *T*_C_^Co^ = *T*_C_^CoFeB^. Here, laser beam (position indicated by the purple circle) sweeps from left to right, with the SFi initialized in State II. The top panel corresponds to σ^−^ laser helicity, bottom to σ^+^. The final magnetic state (shown on the right) is the state at the cross center.

We performed micromagnetic simulations to study the magnetization dynamics induced by the femtosecond laser pulses. Here, as opposed to conventional zero‐temperature simulations, a framework based on the Landau–Lifshitz–Bloch equation is used which is valid for highly nonequilibrium micromagnetism (see Section S5, Supporting Information, for details).^13^ Importantly, this model accounts for all relevant parameters of the experiment, including geometry, magnetic parameters, and thermal and magneto‐optical effects due to the IFE. From simulations we extract values for *V*_Hall_/*V*_Hall_^AP^(*x*), which are in good qualitative agreement with the experimental results (see Section S6, Supporting Information). In **Figure** **3**a, we show simulated snapshots of the transient magnetization in the Hall cross, with *T*_C1_ > *T*_C2_. The trailing edge of the σ^−^ beam restores the initial magnetic state and results in no‐switching (top panel of Figure 3a). For σ^+^, switching occurs (bottom panel of Figure 3a) creating a reversed domain in the illuminated area. The results further validate the proposed switching behavior explanation: The FM layer with the highest *T*_C_ controls the helicity dependence of the SFi ordering.

An interesting property of a SFi is that by appropriate engineering of layer structure one can control the so‐called magnetization compensation point, that is, create a synthetic AF, where *M*_1_ = *M*_2_. This point plays an important role for single‐shot switching in GdFeCo^3^ and could play a similar role in AO‐HDS. To consider this, we first examine Figure 1f, where the critical *t*_CoFeB_ for SFi moment compensation is readily seen. Throughout our measurements the compensation point is systematically at higher *t*_CoFeB_ than the HD inversion thickness, suggesting a limited role of magnetization compensation in this system. Further numerical simulations for parameters at and around the compensation point (varying both *T*_C_ and *M*_S_ *t*_CoFeB_ for the two FMs) also show a dominant role of *T*_c_ in determining AO‐HDS in these SFi's, over any compensation point (see Section S7, Supporting Information).

We further studied the response of the SFi to light pulses with different laser fluence using the sweeping beam method (see Section S8, Supporting Information). In this case, *t*_Co_ = 0.73 nm and *t*_CoFeB_ = 0.54 nm (the switching behavior with a laser fluence of 3.9 mJ cm^−2^ is shown in Figure 1b,c). The results reveal that the demagnetization area (where *V*_Hall_ ≈ 0) reduces as the beam fluence is decreased, effectively indicating that the switching is focused to the center of the beam where the laser provides sufficient energy to create a demagnetized state. With the beam fluence reduced to 1.5 mJ cm^−2^, a minimum in the demagnetization area and subsequent helicity‐dependent switching is observed. Using this laser fluence, the response of the SFi to light pulses using a fixed beam method is also investigated (see Section S9, Supporting Information). The results show that as the laser pulse number increases up to 1 × 10^3^ pulses, *V*_Hall_ decreases toward zero. Partial helicity‐dependent remagnetization occurs when the pulse number exceeds 5 × 10^3^ but, in general, a sweeping beam is required to ensure complete HD‐AOS.

As a final point, we address the origin of the helicity‐independent back switching that occurs when *T*_C1_ ≈ *T*_C2_. When *T*_C1_ = *T*_C2_, the AO‐HDS ring width collapses, resulting in a similar thermal response for each layer. Strong AF coupling remains and consequently no net expansion of one domain structure over the other should take place. Still, a heat gradient is present along the cross arms, due to the localized laser pulse, which is predicted to drive domain walls toward the hotter region (even in ideal antiferromagnets).^16^ The sweeping of the laser spot should therefore cause domain expansion, as DWs propagate along this gradient. In this case, any regions that have not otherwise been demagnetized, for example, the vertical arms of the Hall cross, are expanded, restoring the initial magnetization of the structure. In Figure 3b, we show the calculated transient magnetic configurations in the Hall cross, for the case of *T*_C1_ = *T*_C2_. While in the horizontal wire we obtained a multidomain state, the vertical arm breaks this randomizing effect and restores the initial magnetic state of the cross position. Additional simulations without the vertical arm (see Section S10, Supporting information) show multiple domain states as the laser beam is swept over the wire, confirming that the cross geometry is essential for the backswitching effect.

In addition to explaining AO‐HDS effects in SFi's, the presented results provide valuable information on the AO‐HDS mechanism, in general. Indeed, a picture prevails where the laser pulse must provide sufficient heat for the FM to reach a temperature approaching *T*_C_, at which point light helicity can induce a symmetry‐breaking reversal mechanism, such as the IFE, and lead to switching.

## 3 Conclusion

To summarize, we studied all‐optical helicity‐dependent switching in planar Hall crosses made from rare‐earth free synthetic ferrimagnetic heterostructures (two FM layers, AF exchange coupled). We demonstrate that the AOS not only depends on the light helicity but also on the relative Curie temperature of each ferromagnetic layer. Indeed, by varying the thickness of the individual FM layer constituting the SFi, the helicity dependence of magnetic switching can be modulated. This behavior can be explained by the interaction between the laser polarization and the net moment at switching temperatures, which is governed by the relative *T*_C_ of each layer. When the Curie temperatures of each layer are matched, we observe a helicity‐independent backswitching, with the final magnetic configuration determined only by the initial state. In most materials to‐date helicity dependence is intrinsic, with backswitching demonstrations limited to select rare‐earth alloys,^17^ or numerical calculations.^9^ Here, we successfully demonstrate each of these switching behaviors in Hall devices, made from the same constituent materials and commonly used ferromagnetic materials. Furthermore, Co/Pt and CoFeB/Pt are magnetically high anisotropy materials commonly used in spintronics devices and magnetic recording media. As the current developed synthetic ferrimagnets are composed of these materials, we believe that the developed heterostructures with distinct all‐optical switching behaviors have opened a pathway toward practical opto‐spintronics.

## 4 Experimental Section

*Sample Fabrication*: Samples were grown on glass substrates at room temperature by DC magnetron sputtering, base pressure 6 × 10^−8^ mbar. Two sets of samples were prepared: (a) single FM layers, Ta (3 nm)/Pt (4 nm)/FM_1(2)_/Pt capping (4 nm), where FM_1_ = Co (*t*_Co_ = 0.49 or 0.73 nm), and FM_2_ = CoFeB (*t*_CoFeB_)/Pt (0.4 nm)/CoFeB (*t*_CoFeB_) (0.30 ≤ *t*_CoFeB_ ≤ 0.78 nm). In FM2, both CoFeB layers are FM‐coupled and act as a single FM; (b) SFi samples with structure: Ta (3 nm)/Pt (4 nm)/FM_1_/Pt (0.4 nm)/Ru (0.9 nm)/Pt (0.4 nm)/FM_2_/Pt capping (4 nm). The Pt/Ru/Pt stack provides the antiferromagnetic Ruderman–Kittel–Kasuya–Yosida (RKKY) interlayer exchange coupling between the adjacent FMs.^10^ All layers were grown at 8 × 10^−3^ mbar Ar partial pressure.

*Measurements*: Magnetic properties of the films were studied by vibrating sample magnetometry. The films were patterned into electrical Hall devices by optical lithography and ion milling. The Hall voltage was measured along the *y*‐direction, while a DC current was injected along the *x*‐direction. The width of the current carrying wire is ≈5 µm, giving a DC current density of ≈6 × 10^9^ A m^−2^. A Ti:sapphire fs‐laser was used with wavelength 800 nm, pulse duration 43 fs, and laser repetition rate of 5 kHz. The Gaussian laser beam spot was focused with a full‐width at half‐maximum, FWHM ≈ 46 µm. A quarter‐wave plate was used to create circularly [right‐(σ^+^) and left‐handed (σ^−^)] or linearly polarized (π) beam. The laser fluence is 3.9 mJ cm^−2^, unless otherwise stated, with a half‐wave plate used to adjust beam power. For all experiments, the initial magnetic configuration (state I or II) was set by applying an external saturating magnetic field (±*z*‐direction), before returning to remanence. Hall voltage was constantly monitored (0.2 s per point), and unless stated otherwise, no magnetic field was applied during the measurement. For each initial state, the measurement was repeated using each circular polarization of the incoming light, σ^+^ and σ^−^. Switching was investigated via two methods: (1) Sweeping beam method—the laser beam was swept in discrete steps across the Hall device in the *x*‐direction, at a step rate of 1.4 µm s^‐1^; (2) Fixed beam method—beam position is fixed at the center of the cross, with the number of incident pulses controlled by a pulse picker.

## Conflict of Interest

The authors declare no conflict of interest.

## Supporting information

Supplementary
