# Supplementary material for: Controlling All‐Optical Helicity‐Dependent Switching in Engineered Rare‐Earth Free Synthetic Ferrimagnets
Source: Adv Sci (Weinh). 2019 Oct 14;6(24):1901876. doi: 10.1002/advs.201901876 (PMC6918116; doi:10.1002/advs.201901876)
Supplement: Supplementary file 1 — Supplementary [file ADVS-6-1901876-s001.pdf]

Copyright WILEY-VCH Verlag GmbH & Co. KGaA, 69469 Weinheim, Germany, 2019.

ADVANCED SCIENCE
Open Access

Supporting Information

for *Adv. Sci.,* DOI: 10.1002/advs.201901876

Controlling All-Optical Helicity-Dependent Switching in Engineered Rare-Earth Free Synthetic Ferrimagnets

Jung-Wei Liao, Pierre Vallobra, Liam O'Brien, Unai Atxitia, Victor Raposo, Dorothée Petit, Tarun Vemulkar, Gregory Malinowski, Michel Hehn, Eduardo Martínez, Stéphane Mangin, and Russell P. Cowburn*

Copyright WILEY-VCH Verlag GmbH & Co. KGaA, 69469 Weinheim, Germany, 2019.

Supporting Information

Controlling all-optical helicity dependent switching in engineered rare-earth free synthetic ferrimagnets

Jung-Wei Liao, Pierre Vallobra, Liam O'Brien, Unai Atxitia, Victor Raposo, Dorothée Petit, Tarun Vemulkar, Gregory Malinowski, Michel Hehn, Eduardo Martínez Vecino, Stéphane Mangin, and Russell P Cowburn*

Supporting information 1:
Hall hysteresis loops of the synthetic ferrimagnetic heterostructures

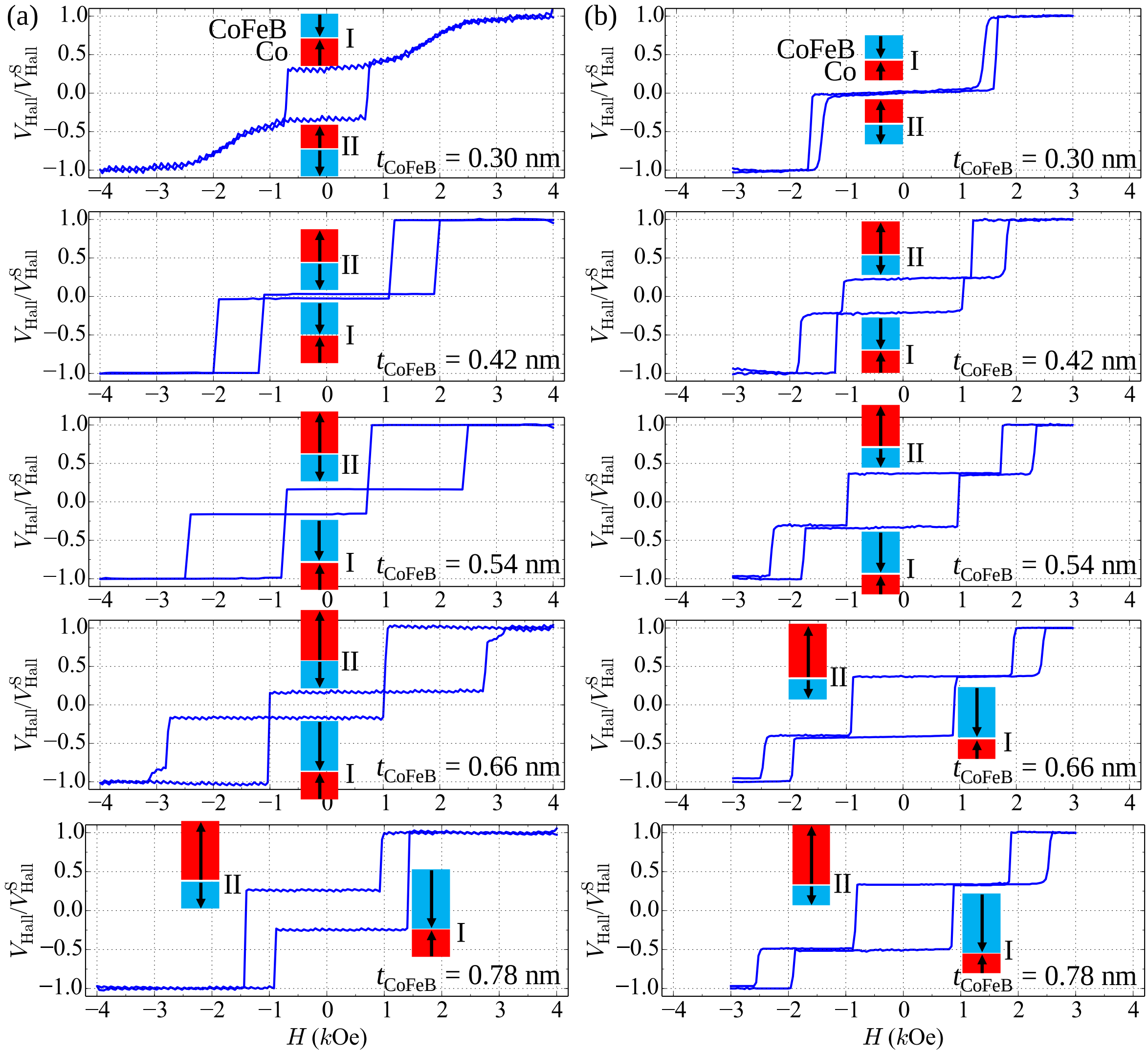

Figure S1. Perpendicular Hall hysteresis loops of the synthetic ferrimagnetic heterostructures composed of two ferromagnetic layers FM_1 and FM_2 with different layer thickness. The layer thickness of FM_1: (a) t_{Co} = 0.73 and (b) 0.49 nm. The layer thickness of FM_2: $0.30 \leq t_{CoFeB} \leq 0.78$ nm. The Hall voltage, V_{Hall}, is normalized to the voltage of the saturation state, V_{Hall}^{S}.

We illustrate the perpendicular Hall hysteresis loops of the synthetic ferrimagnetic structures. The heterostructure is composed of two ferromagnetic layers FM_1 (Co/Pt) and FM_2 $(CoFeB/Pt)_2$. The thickness of the two ferromagnetic layers is different. All heterostructures show remanent magnetic configurations with FM_1 and FM_2 aligned antiparallel. We refer to these two magnetic states as State I (FM_1 magnetization pointing up) and State II (FM_1 magnetization pointing down).

Coercivity, H_C, is measured from a minor loop. For $FM_{1,}$ for t_{Co} = 0.49 nm we obtain H_C ~ 300 Oe, while at t_{Co} = 0.73 nm, H_C ~ 400 Oe. For FM_2, H_C increases from ~ 5 Oe to ~ 480 Oe as t_{CoFeB} increases. As might be anticipated, the Hall voltage change due to FM_2 reversal is found to increase as t_{CoFeB} increases.

Supporting Information 2:

Additional all-optical switching behavior in the synthetic ferrimagnetic heterostructures

2.1: the heterostructure composed of FM1 with t_{Co} = 0.73 nm, and FM_2 with t_{CoFeB} = 0.30 nm

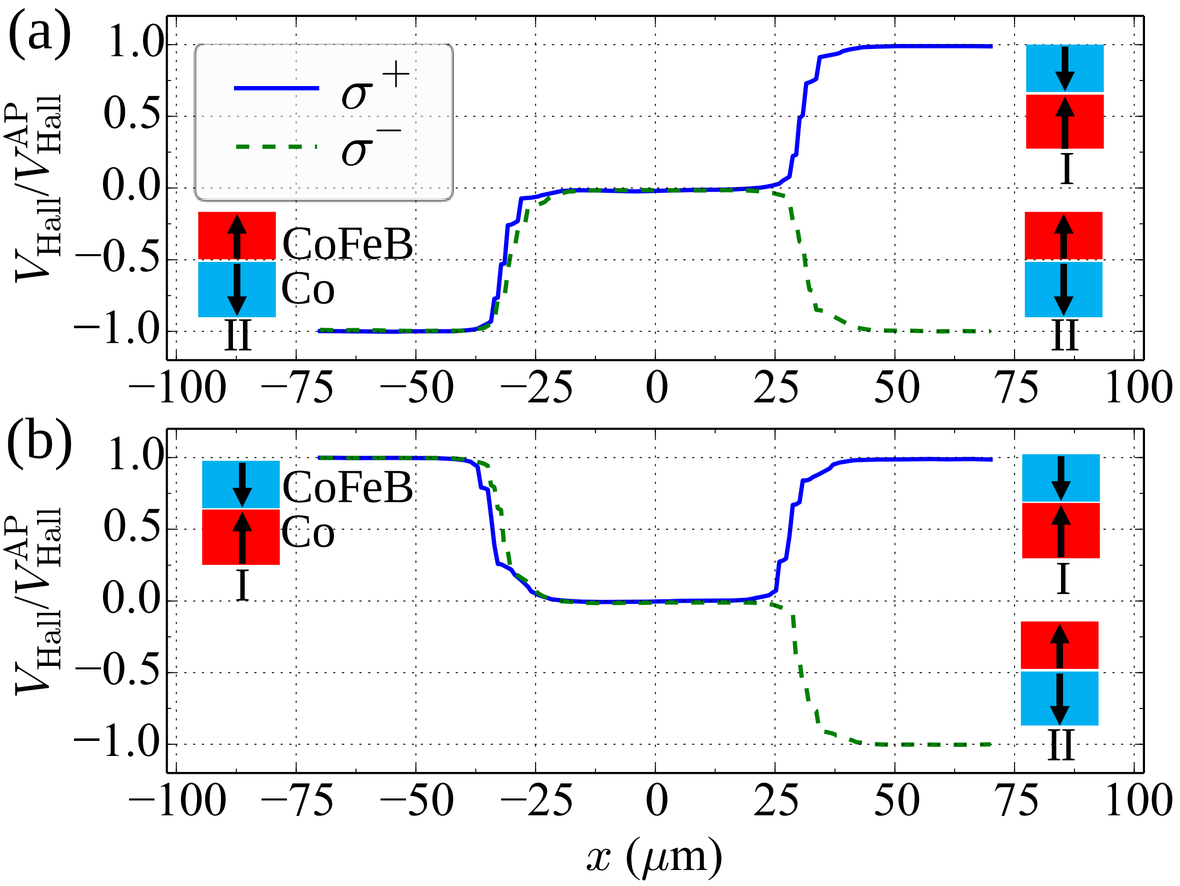

Figure S2.1 Variation of the normalized Hall voltage, V_{Hall}/V_{Hall}^{AP}, as a function of the laser beam position, x. V_{Hall}^{AP} is the Hall voltage of State I or State II. x is the beam position relatively to the center of the Hall cross. The initial state is (a) State II and (b) State I. At each initial state, the measurement was repeated using the beam with two different circular polarizations, σ^+ and σ^-.

All optical switching in the heterostructure with t_{Co} = 0.73 nm and t_{CoFeB} = 0.30 nm is investigated using the laser sweeping method. Figure S2.1 illustrates the variation of the normalized Hall voltage, V_{Hall}/V_{Hall}^{AP}, as a function of the laser beam position, x. All-optical helicity-dependent switching is observed. Using the σ^+ beam leads to the final magnetic configuration of State I, while the σ^- beam gives State II. The helicity dependence of the FM_1 magnetization direction is similar to that of a single FM_1 layer (see Supporting Information 3).

2.2: heterostructure composed of FM_1 with t_{Co} = 0.49 nm, and FM_2 with t_{CoFeB} = 0.54 nm

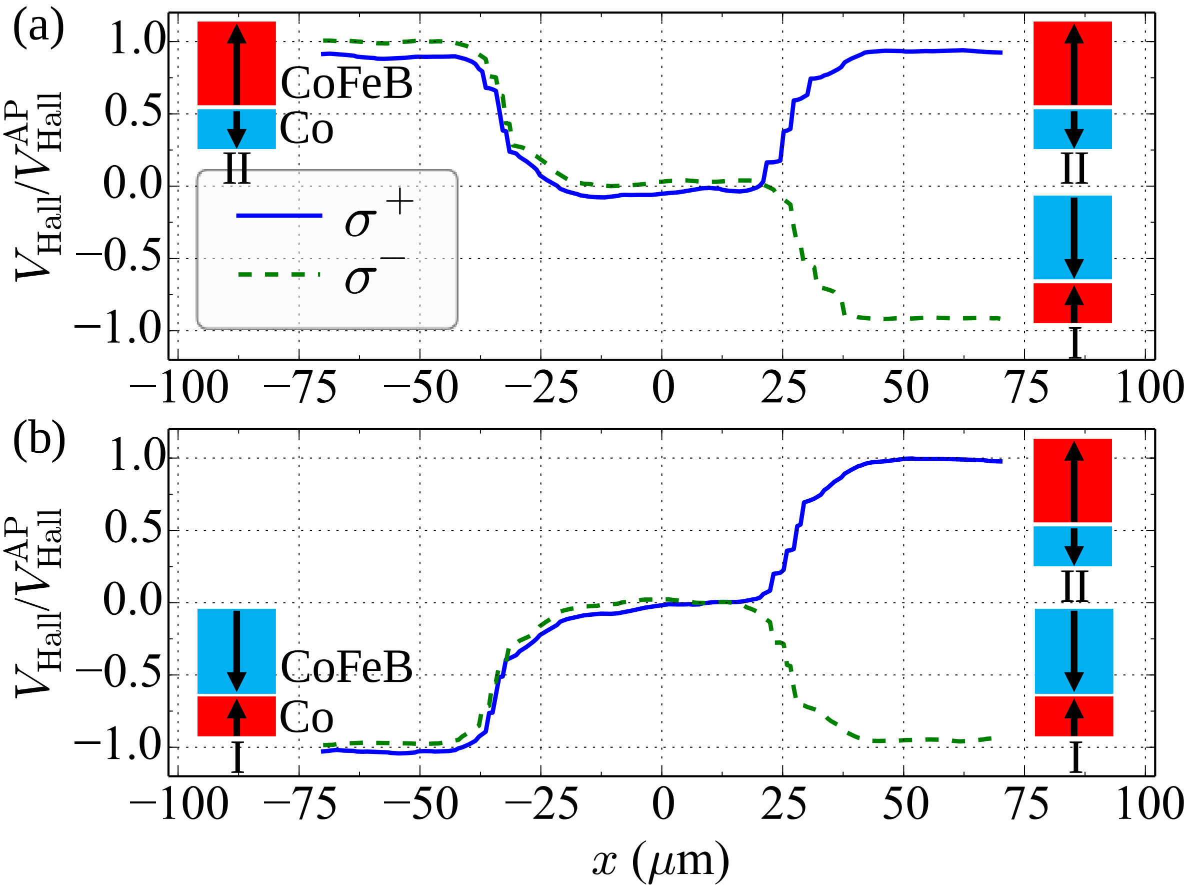

Figure S2.2. Variation of the normalized Hall voltage, V_{Hall}/V_{Hall}^{AP}, as a function of the laser beam position, *x*. V_{Hall}^{AP} is the Hall voltage of State I or State II. *x* is the beam position relatively to the center of the Hall cross. The initial state is (a) State II and (b) State I. At each initial state, the measurement was repeated using the beam with two different circular polarizations, σ^+ and σ^-.

All-optical helicity-dependent switching behavior in the heterostructure with t_{Co} = 0.49 nm and t_{CoFeB} = 0.54 nm, investigated using the laser sweeping method. Figure S2.2 illustrates the variation of the normalized Hall voltage, V_{Hall}/V_{Hall}^{AP}, as a function of the laser beam position, *x*. All-optical helicity-dependent switching is observed. Using the σ^+ beam leads to the final magnetic configuration of State I, while the σ^- beam gives State II. The helicity dependence of the FM_1 magnetization direction is opposite to that in the single FM_1 layer (see Supporting Information 3).

Supporting Information 3:

all-optical helicity-dependent switching in a single Co layer with t_{Co} = 0.73 nm

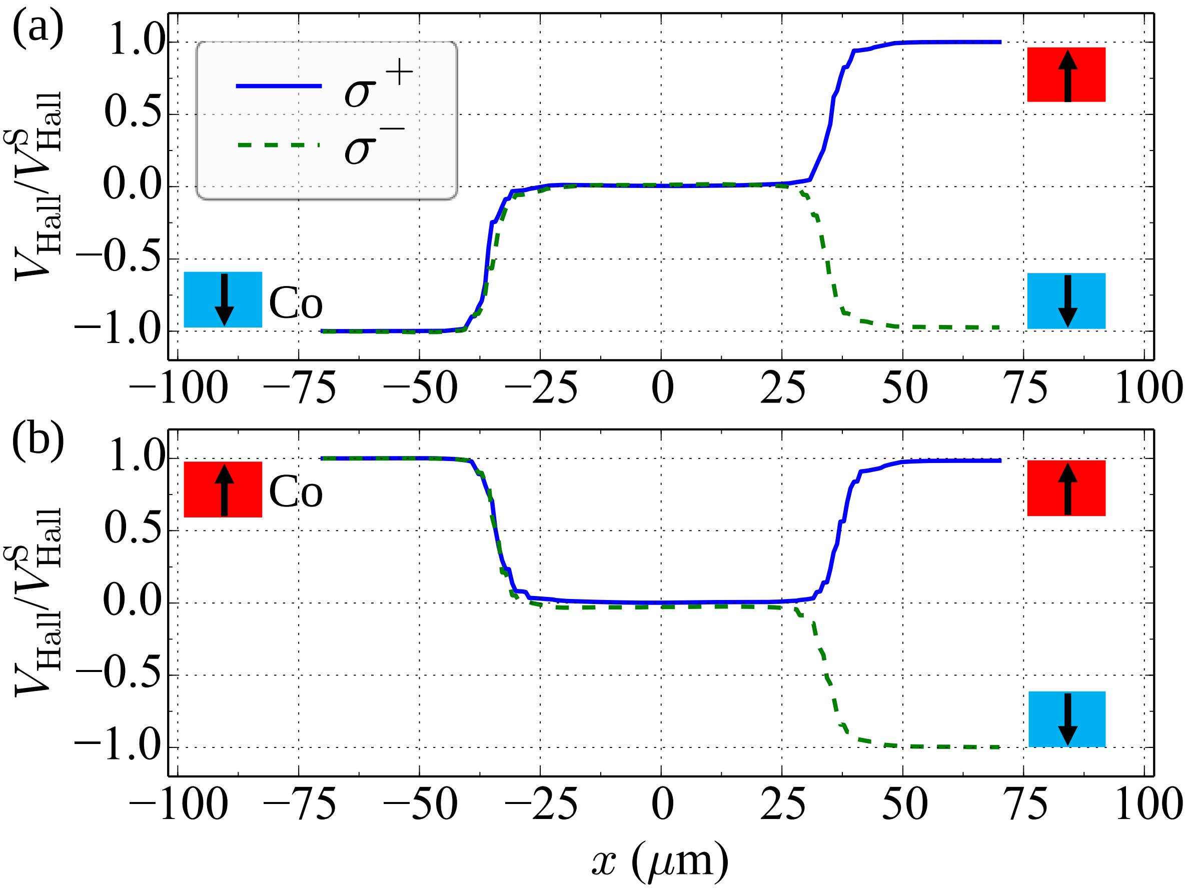

Figure S3. Variation of the normalized Hall voltage, V_{Hall}/V_{Hall}^S, as a function of the laser beam position, x, of the ferromagnetic sample $FM_{1.}$ V_{Hall}^S is the Hall voltage of the saturation state. x is the beam position relative to the center of the cross. The initial magnetic state is (a) magnetization pointing down, and (b) magnetization pointing up. At each initial state, the measurement was repeated using the beam with two different circular polarizations, σ^+ and σ^-.

We investigate the all-optical helicity dependent switching in a single Pt/Co/Pt layer, using the sweeping beam method. Here, t_{Co} = 0.73 nm. Figure S3 show the resulting normalized Hall voltage, V_{Hall}/V_{Hall}^S, as a function of the laser beam position, x. The results show the demagnetization and helicity-dependent re-magnetization processes. Using the σ^+ beam gives the final magnetization state with the magnetization pointing up (+z), while the σ^- beam leads to the state with magnetization pointing down (-z).

Supporting Information 4:

Switching behavior using the linearly polarized beam and an external magnetic field

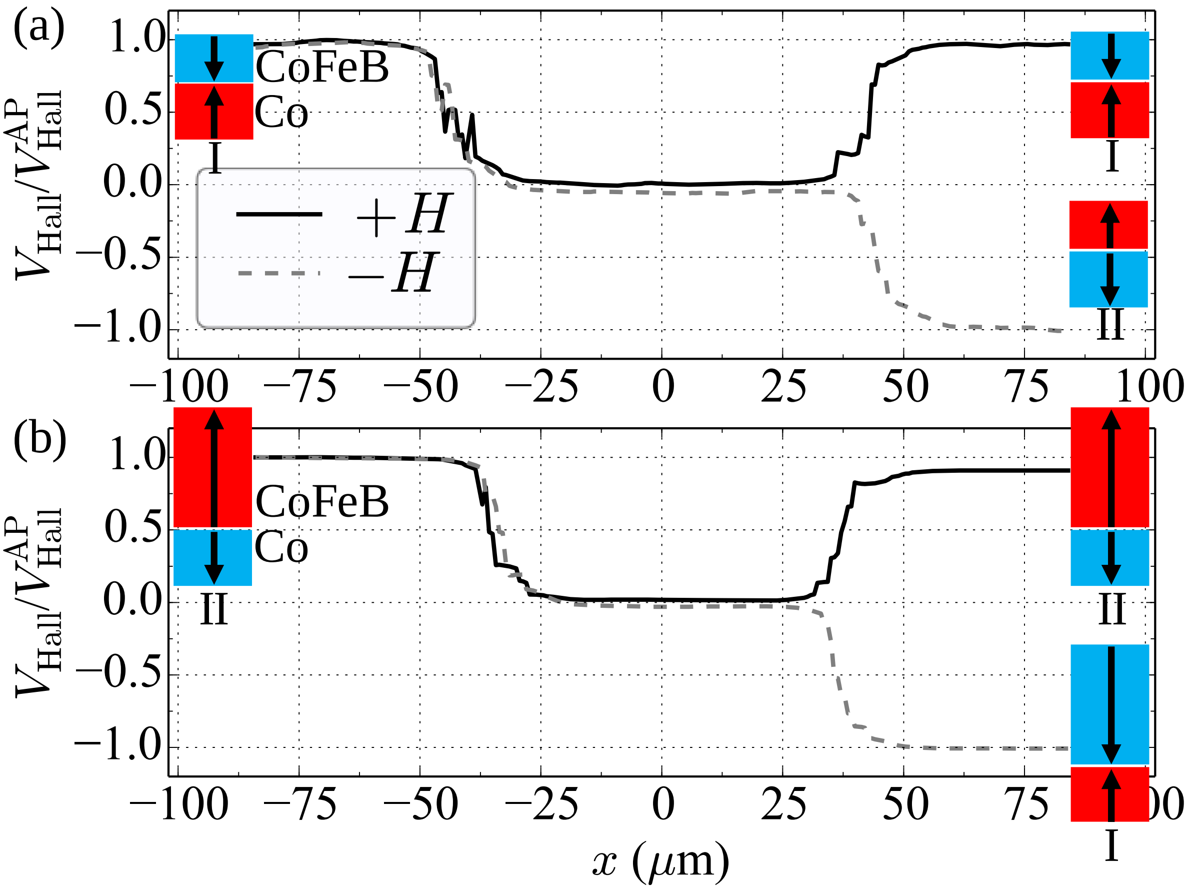

Figure S4. Variation of the normalized Hall voltage, V_{Hall}/V_{Hall}^{AP}, as a function of the laser beam position, *x*. V_{Hall}^{AP} is the Hall voltage of State I or State II. *x* is the beam position relatively to the center of the Hall cross. The heterostructures are made of FM_1 with t_{Co} = 0.73 nm and FM_2 with (a) t_{CoFeB} = 0.30 and (b) t_{CoFeB} = 0.78 nm. The initial state is (a) State I and (b) State II. At each initial state, the measurement was repeated using two different magnetic field directions, +*H* and –*H*. The magnetic field strength is only 3 Oe.

We investigate the switching behavior using a linearly polarized beam and a constant magnetic field. We swept the linearly polarized beam across the Hall cross, while a constant magnetic field, *H*=3 Oe, was applied. We measured the Hall voltage change during this process. At each initial state, we repeated the measurement using the field parallel or antiparallel to the *z*-direction, +*H* and –*H*. We measured two heterostructures, with t_{Co} = 0.73 nm and t_{CoFeB} = 0.30 nm or 0.78 nm. $T_{C1} > T_{C2}$ in the heterostructure with t_{CoFeB} = 0.30 nm, while $T_{C1} < T_{C2}$ in the heterostructure with t_{CoFeB} = 0.78 nm. In Figure S4, we illustrate the resulting normalized Hall voltage, V_{Hall}/V_{Hall}^{AP}, as a function of the laser beam position, *x*.

Under the fixed field direction, the two heterostructures show different final magnetic states. For example, under $+H$ the heterostructure with t_{CoFeB} = 0.30 nm gives State I, where FM_1 magnetization direction is along the field direction [Supplementary Figure 5(a)]. Under the same condition, the heterostructure with t_{CoFeB} = 0.78 nm gives State II with FM_2 magnetization direction along the field direction [Supplementary Figure 5(b)]. The results suggest that the final magnetic state is determined by the magnetic field acting on the FM layer with the highest T_C.

Supporting information 5:

Details of numerical simulation

The micromagnetic magnetization dynamics induced by fs laser pulses are studied by solving the Laudau-Lifshitz-Bloch eq. (LLB) coupled to the Three Temperature Model (3TM), which accounts for the space and temporal evolution of the electron, lattice and spin temperatures of the subsystems. Below, we provide details of the modelled samples and laser beam, as well as the LLB & 3TM models. The micromagnetic results are given at the final part of this section.

Geometry and laser properties

The system under study consists of a multilayer with two ferromagnetic (FM) layers, separated by a spacer, which generates an antiferromagnetic coupling between their local magnetizations. The lower FM layer is on top of a heavy metal (HM), and the upper FM is also under another similar HM layer. The geometry of the evaluated sample is shown in Figure S5.1: a cross with longitudinal (*x*-axis) and transverse (*y*-axis) branches (dimensions indicated in the caption of Figure S5.1).

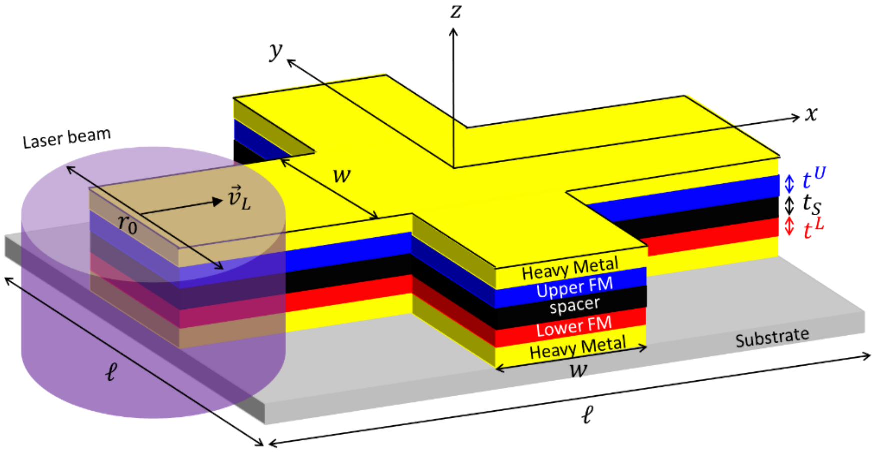

Figure S5.1. Geometry of the studied multilayer with the representation of the laser beam (r_0 is HWHM radius of the laser beam). The dimensions of the simulated sample are defined in the sketch: *l* indicates the in-plane length of the computational region, *w* represents the width of the two orthogonal strips forming the cross, and t^L, t_S and t^U are the thicknesses of the Lower FM, the Spacer and the Upper FM strips respectively. The center of Laser beam is displaced from left to right along the longitudinal *x*-axis with a velocity $\vec{v}_L = v_L\vec{u}_x$, and its HWHM radius is $r_0 = 2w$. The considered dimensions are: $l = 1536$ nm, $w = 192$ nm, $t^L = t_S = t^U = 0.8$ nm, and $v_L = 96$ m/s.

A laser is swept from left to right along the longitudinal (*x*-axis) branch of the cross. The laser spot is assumed to have a Gaussian spatial profile with a full width at half maximum (FWHM) defined by a radius r_0. Laser pulses are incident on the multilayer as the beam moves along the longitudinal branch of the cross. The temporal profile of these pulses is

assumed to be Gaussian, with $\tau_L$denoting the FWHM duration. The power of the laser is consequently:

$$P(\vec{r},t) = P_0 \exp\left[-\frac{|\vec{r}-\vec{r}_L|^2}{r_0^2/(4\ln 2)}\right] \exp\left[-\frac{(t-t_0)^2}{\tau_L^2/(4\ln 2)}\right]$$

where t_0 is the time at which each laser pulse reaches its maximum power (P_0) in the center of the laser spot, or in other words, the central time of the laser pulse, r_0. $\vec{r}_L = \vec{r}_L(t)$ represent the position of the laser and $\vec{r}$ is the position over the sample. As the laser is moving from left to right with constant velocity ($\vec{v}_L = v_L\vec{u}_x$) along the longitudinal x-axis, each laser pulse is applied at different locations, $\vec{r}_L(t) = v_L t\vec{u}_x$ (the center of the laser spot is always focused at the center of the multilayer in the transverse axis, i.e. $y_L = 0$). The maximum power of the laser is $P_0 = F/(t_t\tau_L)$, where F is the laser fluence, and t_t is the total thickness of the multilayer, i.e. $t_t = t^L + t_S + t^U$.

Magnetization dynamics: Laudau-Lifshitz-Bloch eq. (LLB)

The evolution of the local magnetization under these laser pulses is described by the Laudau-Lifshitz-Bloch eq. (LLB). For simplicity, in what follows, we use $\vec{m}$ to denote the local magnetization in each of the two FM layers ($\vec{m}$ represents the local magnetization in the lower FM layer $\vec{m} = \vec{m}^L$, and in the upper FM layer $\vec{m} = \vec{m}^U$). Note that in general, both lower and upper FM layers have different magnetic properties. With this simplification in the notation, the optically-induced magnetization dynamics in each FM is described by the corresponding Laudau-Lifshitz-Bloch eq. (LLB), which is given by

$$\frac{d\vec{m}(\vec{r},t)}{dt} = -\gamma_0'\vec{m}\times\vec{H}_{eff} - \gamma_0\frac{\alpha_\perp}{m^2}\left[\vec{m}\times\left(\vec{m}\times\left(\vec{H}_{eff}+\vec{H}_{th}^{\perp}\right)\right)\right] + \gamma_0'\frac{\alpha_\parallel}{m^2}\left(\vec{m}\cdot\vec{H}_{eff}\right)\vec{m} + \vec{H}_{th}^{\parallel},$$

where $\vec{m}(\vec{r},t) = \vec{M}(\vec{r},t)/M_s^0$ is the normalized magnetization, with M_s^0 the saturation magnetization at $T = 0$, and $m = m(T) \equiv |\vec{m}|$. $\gamma_0' = \gamma_0/(1+\alpha^2)$, where γ_0 is the gyromagnetic ratio and α is the Gilbert damping. $\alpha_\parallel$ and $\alpha_\perp$ are the longitudinal and transverse damping parameters, given by:

$$\alpha_\parallel = \alpha\left(\frac{2T}{3T_C}\right)$$

$$\alpha_\perp = \alpha\left(1-\frac{T}{3T_C}\right),$$

where T_C is the Curie temperature. The effective field $\vec{H}_{eff}$ includes all the conventional interactions of the micromagnetic theoretical framework, and the magneto-optical field due to the Inverse Faraday Effect (IFE).

$\vec{H}_{eff} = \vec{H}_{exch} + \vec{H}_{DM} + \vec{H}_{dmg} + \vec{H}_{ani} + \vec{H}_{int} + \vec{H}_{m} + \vec{H}_{MO}$,

where $\vec{H}_{exch}$ is the exchange contribution, $\vec{H}_{DM}$ is the DMI interaction, $\vec{H}_{dmg}$ is the demagnetizing field, and $\vec{H}_{ani}$ is the magnetic anisotropy. The interlayer exchange coupling ($\vec{H}_{int}$) is also considered. $\vec{H}_{m}$ represents the internal exchange field in the LLB eq, which is given by,

$$\vec{H}_m = \begin{cases} \frac{1}{2\chi_{\parallel}}\left(1 - \frac{m^2}{m_e^2}\right)\vec{m}, & T < T_C \\ -\frac{1}{\chi_{\parallel}}\left(1 + \frac{3}{5}\frac{T_C m^2}{(T - T_C)}\right)\vec{m}, & T > T_C \end{cases}$$

where $\chi_{\parallel}$ is the longitudinal susceptibility,

$\chi_{\parallel} = \left.\frac{\partial m_e}{\partial H_{ext}}\right)_{H_{ext}\to 0}$.

Here, $m_e = m_e(T)$ is the equilibrium value of $m = m(T)$. Note that at a given instant in time, the local magnetization is not, in general, in thermal equilibrium, however, the LLB equation can evaluate this non-equilibrium physics.

The magneto-optical field, $\vec{B}_{MO}(\vec{r}, t) \ = \mu_0 \vec{H}_{MO}(\vec{r}, t)$, is the local effective field due to the circular polarization of the laser beam. This out-of-plane field ($\vec{H}_{MO}$) emerges as consequence of the Inverse Faraday Effect (IFE), and can be expressed as:

$\vec{B}_{MO}(\vec{r}, t) = (\sigma^{\pm})\ F\ \chi_{IFE}\ f_{MO}(\vec{r}, t)\vec{u}_z$,

where $\sigma^{\pm} = \pm 1$ is the laser helicity, F (in $[(\mathrm{J/m^2})]$) is the laser fluence and χ_{IFE} (in $[\mathrm{T/(J/m^2)}]$) is the inverse Faraday effect susceptibility. Therefore, the maximum value of the magneto-optical field ($B_{max} = F\ \chi_{IFE}$) is reached when the laser power reaches its maximum value, at the center of the laser beam. The spatio-temporal dependence of the magneto-optical field is described by the function $f_{MO}(\vec{r}, t)$

$$f_{MO}(\vec{r}, t) = \begin{cases} \exp\left[-\frac{|\vec{r} - \vec{r}_L|^2}{r_0^2/(4\ln 2)}\right]\exp\left[-\frac{(t - t_0)^2}{\tau_L^2/(4\ln 2)}\right], & t < t_i \\ \exp\left[-\frac{|\vec{r} - \vec{r}_L|^2}{r_0^2/(4\ln 2)}\right]\exp\left[-\frac{(t - t_0)^2}{(\tau_L + \tau_d)^2/(4\ln 2)}\right], & t > ti \end{cases}$$

where t_0 is the time at which each laser pulse reaches its maximum power (P_0) in the center of the laser spot, or in other words, the central time of the laser pulse, r_0. $\vec{r}_L = \vec{r}_L(t)$ represent

the position of the laser and $\vec{r}$ is the position over the sample. τ_d represents the delay of the magneto-optical field with respect to the laser pulse and, in agreement with several experimental observations, it accounts for some persistence of the magnetic field due to the optical signal. The temporal evolution of the normalized magneto-optical field (B_{MO}/B_{max}) and the normalized laser power (P/P_{max} where $P_{max} \equiv P_0$) at the center of the laser beam are both shown in Figure S5.2, where the laser pulse duration is $\tau_L = 200$ fs, and the delay of the magneto-optical field is $\tau_d = \tau_L$ (here σ^+ is used). The fluence considered is $F = 6\ \mathrm{J/m^2}$. Note that the spatial dependence of the magneto-optical field is similar to that of the laser power, *i.e.* the magneto-optical field is Gaussian with a full width at half maximum (FWHM) defined by the radius r_0.

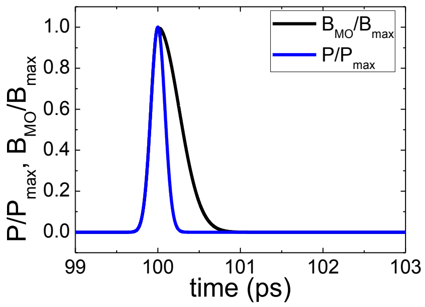

Figure S5.2. Laser beam and magneto-optical field properties. Temporal evolution of the normalized magneto-optical field (B_{MO}/B_{max}) and the normalized laser power (P/P_{max} where $P_{max} \equiv P_0$) at the center of the laser beam. The laser pulse is $\tau_L = 200$ fs, and the delay of the magneto-optical field is $\tau_d = \tau_L$ (with σ^+). The fluence considered here is $F = 6\ \mathrm{J/m^2}$.

The LLB eq. also includes stochastic terms $\vec{H}_{th}^{\perp}$ and $\vec{H}_{th}^{\parallel}$ to account for stochastic fluctuations due to the thermal noise. The first one ($\vec{H}_{th}^{\perp}$) is a random thermal field orthogonal to the local magnetization, while the second one ($\vec{H}_{th}^{\parallel}$) describes the longitudinal noise, parallel to the local magnetization. Their statistical properties are summarized by:

$$\langle H_i^{\perp}(t)\rangle = 0$$

$$\langle H_i^{\perp}(\vec{r},t)H_j^{\perp}(\vec{r}',t')\rangle = \frac{2K_B T(\alpha_\perp - \alpha_\parallel)}{\gamma_0' M_s^0 V \alpha_\perp^2}\delta_{ij}\delta(t-t')\delta(\vec{r}-\vec{r}')$$

$$\langle H_i^{\parallel}(t)\rangle = 0$$

$$\langle H_i^{\parallel}(\vec{r},t)H_j^{\parallel}(\vec{r}',t')\rangle = \frac{2\gamma_0' T\alpha_\parallel}{M_s^0 V\alpha_\perp^2}\delta_{ij}\delta(t-t')\delta(\vec{r}-\vec{r}')$$

$$\langle H_i^{\perp}(\vec{r},t)H_j^{\parallel}(\vec{r}',t')\rangle = 0$$

In these expressions, the notation $\langle \cdots \rangle$ indicates the average over different stochastic realizations of the noise. K_B is the Boltzmann constant and V is the volume of the computational cell.

Disorder: In order to account for the effects of disorder (e.g. due to imperfections and defects) in a realistic way, we assume that the easy axis anisotropy direction ($\vec{u}_K = \vec{u}_K(\vec{r}_G)$) is distributed among a length scale defined by the grain size. The average size of the grains is taken to be 10 nm. Despite the fact that the direction of the uniaxial anisotropy of each grain is mainly directed along the perpendicular direction (z-axis), a small in-plane component, less than 5%, is randomly generated over the grains. Although other ways to account for imperfections could be adopted, we selected this one based on previous studies, which properly describe other experimental observations.

Magnetic parameters: The magnetic parameters considered in the study are typical of Pt/Co multilayers. For simplicity, we assume that the exchange stiffness parameter, $A_{ex} = 1.5 \times 10^{-11}\mathrm{J/m}$, the perpendicular magnetic anisotropy constant, $K_u = 1.25 \times 10^6\ \mathrm{J/m^3}$, and the Gilbert damping, $\alpha = 0.5$, are common parameters for the lower and the upper FM layers. The saturation magnetization (M_S^L, M_S^U) and the Curie temperature (T_C^L, T_C^U) vary for the lower and the upper FM layers, independently. Note, the *Tc* values are chosen to be comparable to that observed in experiment. The DMI parameters are $D^L = +1.25\ \mathrm{mJ/m^2}$ and $D^U = -1.25\ \mathrm{mJ/m^2}$, and the interlayer exchange constant is $J^{ex} = -0.35\ \mathrm{mJ/m^2}$. The magneto-optical field induced by the laser pulses is assumed the same in both layers, with $F\chi_{IFE} = 5\ \mathrm{T}$.

Temperature dynamics: Three Temperature Model (3TM)

The temperature evolution in the system, under the action of the laser pulses, can be described in terms of three subsystems, characterized by the electron (T_e), lattice (T_l) and spin ($T_s \equiv T$) temperatures. Note that the relevant temperature for the magnetic system, described by the previously introduced LLB eq., is the spin temperature, $T_s(\vec{r},t) \equiv T(\vec{r},t)$, but this will ultimately depend on the temperature of both the electron ($T_e(\vec{r},t)$) and lattice ($T_l(\vec{r},t)$) subsystems, as given by the following coupled set of differential equations:

$$\begin{cases} C_e \frac{\partial T_e}{\partial t} = -k_e \nabla^2 T_e - g_{el}(T_e - T_l) - g_{es}(T_e - T_s) + P(\vec{r}, t) \\ C_l \frac{\partial T_l}{\partial t} = -k_l \nabla^2 T_l - g_{el}(T_l - T_e) - g_{ls}(T_l - T_s) \\ C_s \frac{\partial T_s}{\partial t} = -k_s \nabla^2 T_s - g_{es}(T_s - T_e) - g_{ls}(T_s - T_l) \end{cases}.$$

Here, C_i is the thermal capacity (in $[\mathrm{J/(m^3K)}]$) and k_i is the thermal conductivity (in $[\mathrm{W/(m \cdot K)}]$) of each system ($i: e, l, s$). g_{ij} are the coupling constants between subsystems (in $[\mathrm{W/K}]$) and $P(\vec{r}, t)$ is the laser power. Note that above the Debye temperature, C_l and C_s can be considered as constant parameters, whereas, C_e is linear with the electron's temperature, $C_e = \gamma_e T_e$, with $\gamma_e = \frac{C_e(300\ \mathrm{K})}{300\ \mathrm{K}}$. The influence of the substrate can be also taken into account by adding an additional Newton-like term, $(-(T - T_{sub})/\tau_{sub})$, to the right-hand side of equation 2 in the coupled set. In this case, T_{sub} is the substrate temperature, and τ_{sub} is a characteristic time describing the heat transport to the substrate and the surrounding.

Thermal parameters: The following thermal parameters found the literature were considered in the present micromagnetic analysis: $C_e(300\ \mathrm{K}) = \gamma_e T_e = 2.8 \times 10^5\ \mathrm{J/(m^3K)}$ at $T_e = 300\ \mathrm{K}$, $C_l = 2.8 \times 10^5\ \mathrm{J/(m^3K)}$, $C_s = 2.8 \times 10^5\ \mathrm{J/(m^3K)}$, $k_e = 91\ \mathrm{W/(m^3 \cdot K)}$, $k_l = k_s = 0$, $g_{ij} = 1.5 \times 10^{18}\ \mathrm{W/m^3}$, $T_{sub} = 300\ \mathrm{K}$, and $\tau_{sub} = 0.09$ ns.

By solving the 3TM equations with these typical parameters, it is easy to see that under the application of a short laser pulse with ~100 fs, the 3TM equations predict an abrupt increase in the electron (T_e) and spin (T_S) temperatures, followed by a decrease (or relaxation) towards room temperature. Figure S5.3 shows an example of these temperature dynamics for τ_L = 200 fs. Note, the lattice temperature (T_l) increases relatively slowly. After the laser pulse, the three temperatures tend towards the same limiting value, with a characteristic time of a few ~ 1 ps. Note that on these time scales, the diffusive terms ($\nabla^2 T_i$) are negligible.

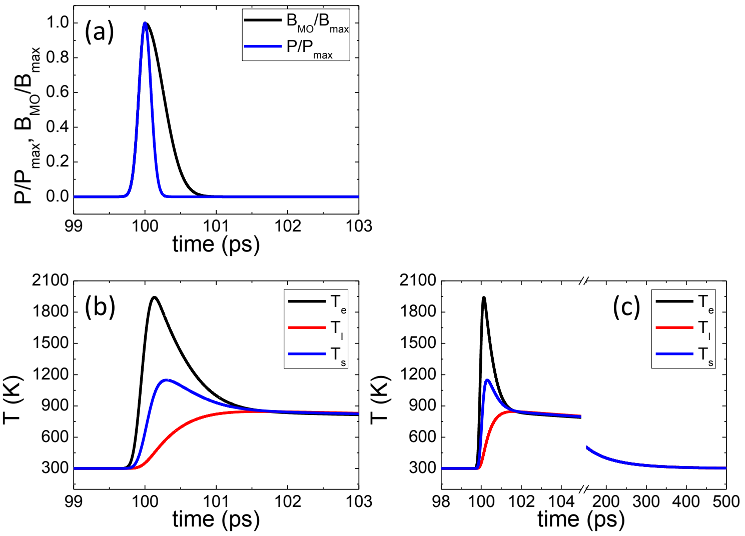

Figure S5.3. Temporal evolution of the temperatures of the three subsystems (electrons, lattice and spins) in the center of the laser beam, as obtained from the 3TM. (a) Temporal evolution of the normalized magneto-optical field (B_{MO}/B_{max}) and the normalized laser power (P/P_{max} where $P_{max} \equiv P_0$) at the center of the laser beam. The laser pulse is τ_L = 200 fs, and the delay of the magneto-optical field is $\tau_d = \tau_L$ (with σ^+). (b) Temporal evolution of the temperature of the three subsystems: electrons (T_e), lattice (T_l) and spins (T_S). These temperatures are evaluated at the center of the laser beam. (c) Same as in (b) with indication of the relaxation of the three temperatures towards room value for longer times. The fluence considered here is F = 6 J/m^2.

Numerical details: The FM layers and the spacer were discretized using a 3D finite difference scheme. The in-plane size of the computational cells is $\Delta x = \Delta y$ = 3 nm, while Δz = 0.8 nm, which coincides with the thickness of the FM layers and the spacer. The dimensions of the cross hall are given in Figure 1. The center of the laser beam is displaced from the left to the right along the longitudinal x-axis with a velocity v_L = 96 nm/ns. A laser pulse is applied every 1 ns, and therefore, 18 laser pulses are applied to cover the longitudinal axis of the cross, from left (x = -768 nm) to right (x = +768 nm). Micromagnetic simulations were performed with an adaptive time step: during each laser pulse, which is τ_L = 200 fs long, the time step is set at Δt = 1 fs. Once each laser pulse is turned off, the time step was enlarged to Δt = 25 fs, to speed up computation. The delay of the magneto-optical field with respect to the laser pulse is $\tau_{MO} = 2\tau_L$. Several tests were performed with reduced cell sizes and time steps to ensure the numerical validity of the presented results.

Supporting information 6:

Micromagnetic simulation results: role of different ferromagnetic layer Curie temperature

Table S1. Curie temperature and saturation magnetization values of the bottom (T_C^{Co} and M_S^{Co}) and the top ferromagnetic layers (T_C^{CoFeB} and M_S^{CoFeB}) of the three simulated bilayer structures, with a Hall cross geometry.

	T_C^{Co} (K)	T_C^{CoFeB} (K)	M_S^{Co} (emu/cc)	M_S^{CoFeB} (emu/cc)
sample a	550	450	1100	1000
sample b	550	550	1100	1100
sample c	550	650	1100	1200

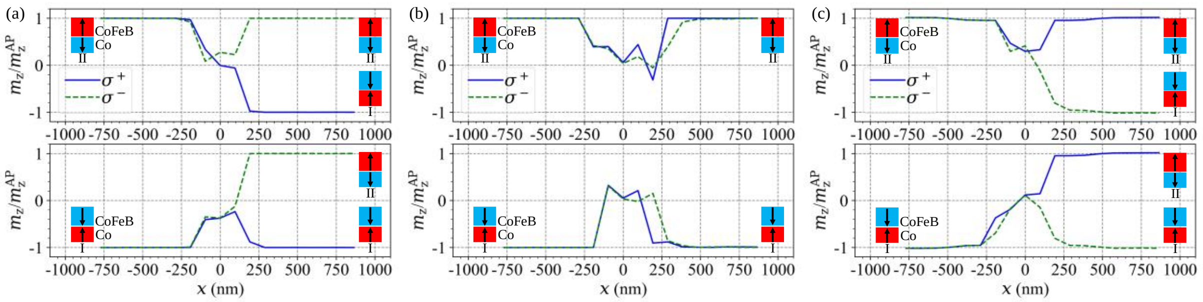

Figure S6. (a) – (c) Micromagnetic simulation presenting the variation of the normalized out-of-plane net magnetization averaged over the center square of the Hall cross, m_z/m_z^{AP}(x), of the synthetic ferrimagnetic heterostructures composed of FM_1 and FM_2 with (a) $T_C^{Co} > T_C^{CoFeB}$, $M_S^{Co} > M_S^{CoFeB}$, (b) $T_C^{Co} = T_C^{CoFeB}$, $M_S^{Co} = M_S^{CoFeB}$, and (c) $T_C^{Co} < T_C^{CoFeB}$, and $M_S^{Co} < M_S^{CoFeB}$. m_z^{AP} is the net magnetization of State II, and x is the laser position. The initial state is State I or State II. The simulations are repeated using the laser beam with two different circular polarizations, σ^+ and σ^-.

We performed micromagnetic simulations for three different bilayers with varying Tc for both the bottom FM layer, T_C^{Co}, and that of the top ferromagnetic layer, T_C^{CoFeB}. The corresponding Curie temperature and the saturation magnetization values of the three calculated bilayers (samples a – c) are enumerated in Table I. We note that, differently to the experimental bilayers, here we have fixed the layer thickness of both FM1 and FM2 to save the computational effort. To appropriately consider the change in magnetization per unit area of FM2, we instead change the M_S value of FM2 (M_S^{CoFeB}). To compare the experimental results of the normal Hall voltage as a function of the laser position, we have calculated the perpendicular magnetization averaged over the center square of the Hall cross, normalizing the net magnetization to the magnetization of State II (FM1 magnetization pointing down, FM2 magnetization pointing up). Figure S6 shows the resulting normalized magnetization as a function of the laser position (x). The simulated results are in good qualitative agreement with the experimental observations, e.g., Figure 2 (a) – (c).

Supporting information 7:

Micromagnetic simulation results: effect of different Curie temperatures and perpendicular magnetization per unit area of two ferromagnetic layers

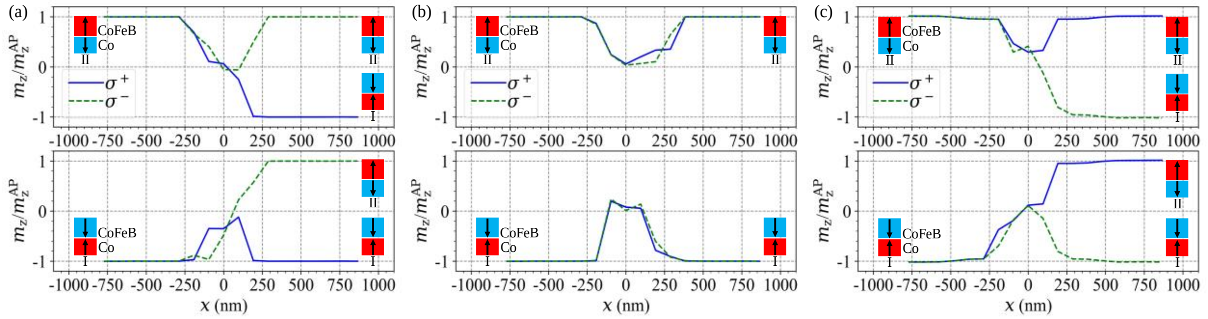

Figure S7.1 (a) – (c) Micromagnetic simulation presenting the variation of the normalized out-of-plane net magnetization averaged over the center square of the Hall cross, m_z/m_z^{AP}(x), of the synthetic ferrimagnetic heterostructures composed of FM_1 and FM_2 with (a) $T_C^{Co} > T_C^{CoFeB}$, (b) $T_C^{Co} = T_C^{CoFeB}$, and (c) $T_C^{Co} < T_C^{CoFeB}$. For all three bilayers, $M_S^{Co} < M_S^{CoFeB}$. m_z^{AP} is the net magnetization of State II, and x is the laser position. The initial state is State I or State II. The simulations are repeated using the laser beam with two different circular polarizations, σ^+ and σ^-.

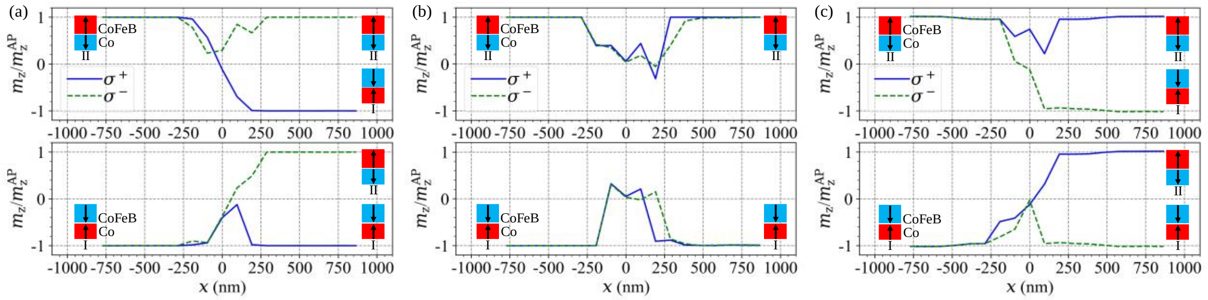

Figure S7.2 (a) – (c) Micromagnetic simulation presenting the variation of the normalized out-of-plane net magnetization averaged over the center square of the Hall cross, m_z/m_z^{AP}(x), of the synthetic ferrimagnetic heterostructures composed of FM_1 and FM_2 with (a) $T_C^{Co} > T_C^{CoFeB}$, (b) $T_C^{Co} = T_C^{CoFeB}$, and (c) $T_C^{Co} < T_C^{CoFeB}$. For all three bilayers, $M_S^{Co} = M_S^{CoFeB}$. m_z^{AP} is the net magnetization of State II, and x is the laser position. The initial state is State I or State II. The simulations are repeated using the laser beam with two different circular polarizations, σ^+ and σ^-.

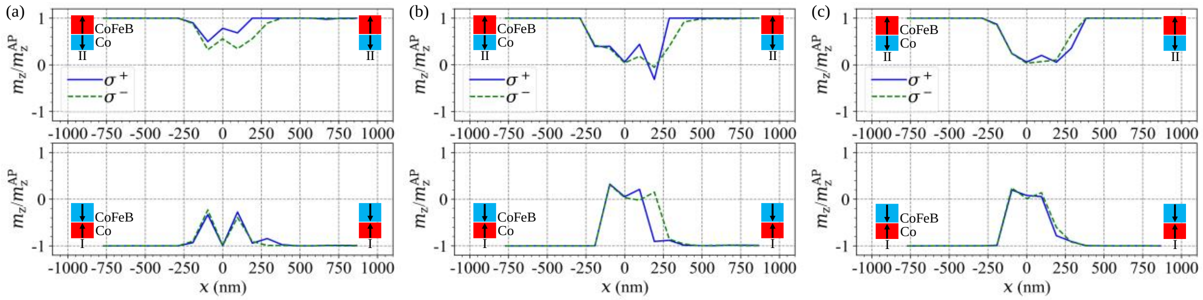

Figure S7.3 (a) – (c) Variation of the normalized out-of-plane net magnetization averaged over the center square of the Hall cross, $m_z/m_z^{AP}(x)$, of the synthetic ferrimagnetic heterostructures composed of FM_1 and FM_2 with (a) $M_S^{Co} > M_S^{CoFeB}$, (b) $M_S^{Co} = M_S^{CoFeB}$, and (c) $M_S^{Co} < M_S^{CoFeB}$. For all three bilayers, $T_C^{Co} = T_C^{CoFeB}$. m_z^{AP} is the net magnetization of State II, and *x* is the laser position. The initial state is State I or State II. The simulations are repeated using the laser beam with two different circular polarizations, σ^+ and σ^-.

To further understand any effects due to changes in saturation magnetization per unit area, we performed additional simulations with different combinations of the saturation magnetization and the Curie temperature values. Figure S7.1 show the results of three SFi bilayers with a fixed difference between the saturation magnetization of the two ferromagnetic layers (i.e. $M_S^{Co} < M_S^{CoFeB}$) but with varying Curie temperature ($T_C^{Co} > T_C^{CoFeB}$, $T_C^{Co} = T_C^{CoFeB}$, and $T_C^{Co} < T_C^{CoFeB}$). The switching behavior agrees with the experiment observations: the FM layer with the highest Curie temperature dominates the helicity dependency. Figure S7.2 show the results of the three bilayers with the same saturation magnetization ($M_S^{Co} = M_S^{CoFeB}$) but different Curie temperature. Once again, the relative Tc values dictate final state, suggesting that any changes to the saturation magnetization are largely irrelevant for the different switching behavior. Finally, we have performed the calculations in the three bilayers with the same Curie temperature ($T_C^{Co} = T_C^{CoFeB}$) but varying saturation magnetization values, as shown in Figure. S7.3. All three bilayers show helicity-*independent* back switching behavior, regardless of different saturation magnetization values. Therefore, we conclude that FM layers with different Curie temperatures are a key ingredient to determine the switching behavior.

Supporting information 8

Different laser fluence

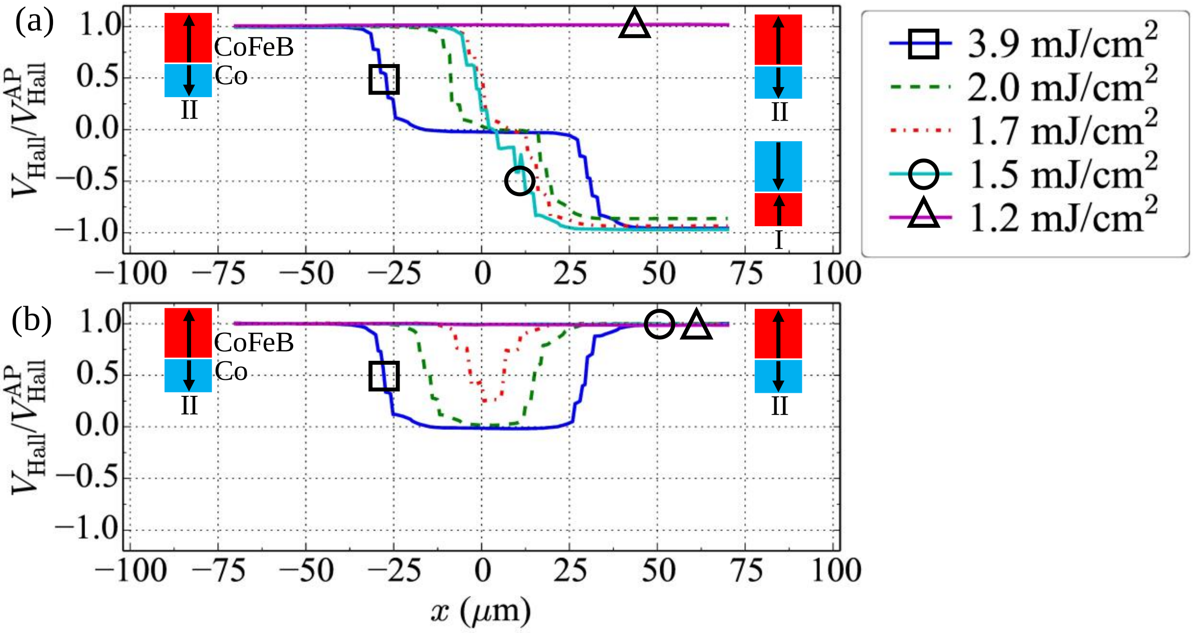

Figure S8. Measured normalized Hall voltage, V_{Hall}/V_{Hall}^{AP}, vs beam position, x (relative to cross center) with different laser fluence for an SFi Hall bar with t_{Co} = 0.73 nm and t_{CoFeB} = 0.54 nm. The measurements are repeated with two different circular polarizations, (a) σ^+, and (b) σ^-. The initial magnetic state is State II.

Response of the SFi, with t_{Co} = 0.73 nm and t_{CoFeB} = 0.54 nm, to fs light pulses with different laser fluence is investigated using the sweeping laser beam method. The SFi was initialized in State II before pulsed, circularly-polarized laser light is swept along the x-axis arm. Figure S.8 (a) (with the incident laser polarization σ^+) and (b) (with the incident laser polarization σ^-) illustrate the resulting V_{Hall} vs relative beam position, x, for each laser fluence. As the laser spot passes the cross, thermal demagnetization and helicity-dependent switching is consistently observed when the average laser fluence >1.7 mJ/cm^2. As the average laser fluence is reduced, the region over which demagnetization is observed (V_{Hall}(x) ~ 0) decreases and eventually disappears. Since the laser energy is distributed in a Gaussian profile, with a larger fluence at the center, our measurements are consistent with the existence of two energy thresholds: 1) the low fluence regime where no switching effect is observed, 2) the intermediate regime when All Optical Helicity Dependent Switching is observed, 3) the high energy regime leading to demagnetization.

Supporting information 9

Fixed laser position with different numbers of laser pulses

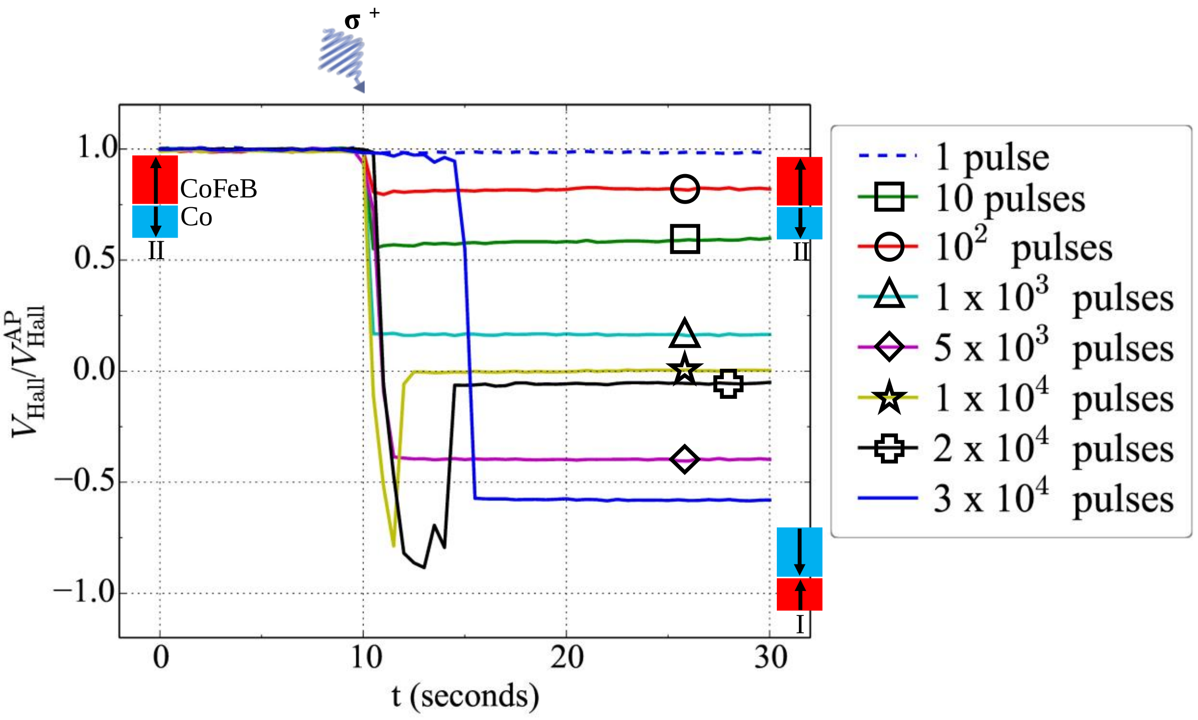

Figure S9. Measured normalized Hall voltage, V_{Hall}/V_{Hall}^{AP}, vs numbers of laser pulses for an SFi Hall bar with t_{Co} = 0.73 nm and t_{CoFeB} = 0.54 nm. The laser beam position is fixed at the center of the Hall bar (x = 0 µm). The initial magnetic state is the state II. The laser pulses were set at time t = 10 seconds, and the normalized Hall voltage was constantly monitored. The laser fluence is 1.5 mJ/cm^2.

Using the fixed beam method, the response of the SFi (t_{Co} = 0.73 nm and t_{CoFeB} = 0.54 nm) to fs light pulses is investigated. The beam position is fixed at the center of the cross, and different numbers of incident pulses were applied while V_{Hall} is monitored. The initial state of the SFi is set in State II, with the beam circular polarization fixed as σ^+ and the laser fluence set as 1.5 mJ/cm^2. Previously, with the laser sweeping method this condition gives the minimum fluence for switching from State II to State I (Figure 1). Figure S9 shows the resulting V_{Hall} vs time t (before and) after illuminating the Hall device. Here, a varying number of laser pulses were applied, commencing at t = 10 s. As the laser pulse number increases up to 1x10^3 pulses, V_{Hall} decreases towards to zero. Partial re-magnetization occurs (with -1.0 < V_{Hall}/V_{Hall}^{AP} < 0) when the pulse number > 5 x 10^3. We note that, with the beam fluence increased to 3.9 mJ/cm^2, V_{Hall}/V_{Hall}^{AP} ~ 0 for all pulse numbers (not shown).

Supporting information 10

Micromagnetic simulation details of bilayers with a wire geometry

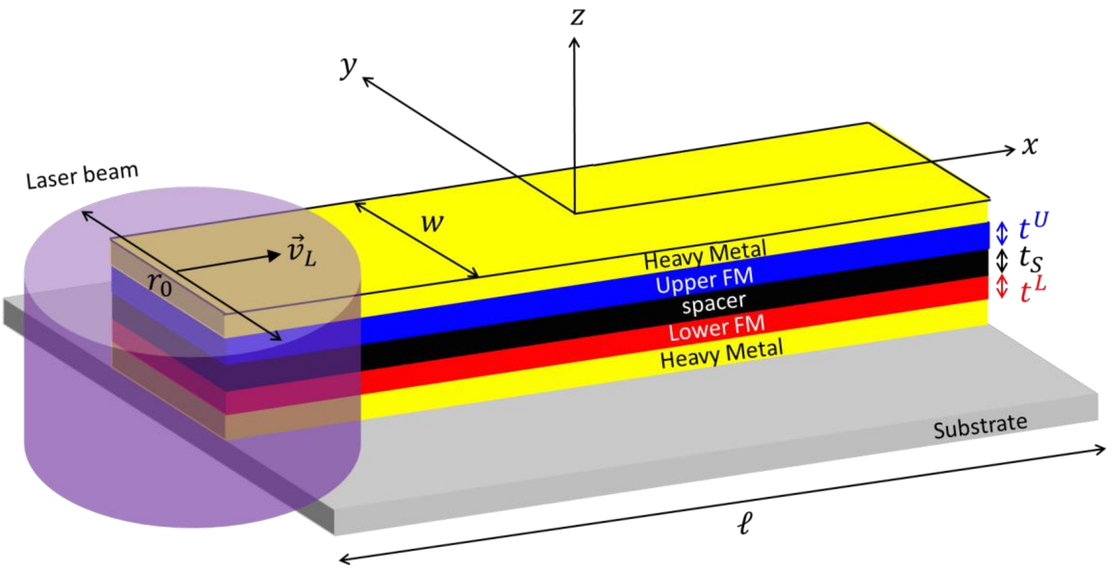

Figure S10. Wire geometry of the studied multilayer with the representation of the laser beam (r_o is HWHM radius of the laser beam). The dimensions of the simulated sample are defined in the sketch: l indicates the in-plane length of the computational region, w represents the width of the two orthogonal strips forming the cross, and t^L, t_S and t^U are the thicknesses of the lower FM, the spacer and the upper FM strips respectively. The center of laser beam is displaced from left to right along the longitudinal x-axis with a velocity v_L, and its FWHM radius is r_o=2w. The considered dimensions are: l = 1536 nm, w = 192 nm, $t^L = t_S = t^U$=0.8 nm, and v_L=96 m/s.

In order to understand the role of the Hall cross geometry on the helicity-independent back switching behavior, in the main article we discuss additional simulations, considering a straight wire geometry. Figure S10 shows a schematic diagram of this case. We note that, for this designed geometry, all areas are illuminated by laser beams during calculations.
